# Histological Evaluation of Wound Healing Process after Photodynamic Therapy of Rat Oral Mucosal Ulcer

**Published:** 2016-03

**Authors:** Parviz Deyhimi, Heidar Khademi, Reza Birang, Mohammad Akhoondzadeh

**Affiliations:** 1 Torabinejad Dental Research Center, Dept. of Oral and Maxillofacial Pathology, School of Dentistry, Isfahan University of Medical Sciences, Isfahan, Iran.; 2 Torabinejad Dental Research Center, Dept. of Oral and Maxillofacial diseases, School of Dentistry, Isfahan University of Medical Sciences, Isfahan, Iran.; 3 Torabinejad Dental Research Center, Dept. of Periodontology, School of Dentistry, Isfahan University of Medical Sciences, Isfahan, Iran.; 4 Dept. of Oral and Maxillofacial Diseases, School of Dentistry, Boushehr University of Medical Sciences, Boushehr, Iran.

**Keywords:** Laser, Methylene Blue, Photodynamic Therapy, Wound Healing

## Abstract

**Statement of the Problem:**

When the body defense is compromised, wounds can act as a route for entrance and colonization of microorganisms in the body. Photodynamic therapy with methylene blue is known as a promising antimicrobial modality.

**Purpose:**

The present study aimed to investigate the effects of this procedure on wound healing processes.

**Materials and Method:**

In this experimental study, 48 male Wistar rats were recruited. Experimental wounds were surgically made on their buccal mucosa. Based on the treatment modality, they were divided into 3 groups (n=16) of control (CG), laser (LG), photosensitizer+ laser (PLG) by methylene blue (MB). The treatment procedure in the two latter groups was done in days 1-4 and 6-9. After sacrificing on 2, 4, 7 and 14-day follow-ups, the microscopic grade of healing of the wounds was assigned on each interval according to histological grading criteria.

**Results:**

A qualitative result was obtained that showed a healing progression in PLG at day 2 of follow-up. At day 4 of follow-up, no difference was seen in healing stage among the groups. However on day 7 of follow-up, samples of the LG showed a lower degree of healing compared with the other two groups. Likewise, on day 14 of follow- up, both PLG and LG showed lower degree of healing than CG.

**Conclusion:**

This study qualitatively showed that MB- mediated photodynamic therapy would have an inhibitory effect on healing process after 14 days of the wound creation.

## Introduction

The oral mucosa is a well-adapted structure to protect the underlying tissues against mechanical damage and inhibits the entry of microorganisms and their toxic materials. However, this protective barrier is disrupted by wound formation which appears as a complication of some diseases such as diabetes mellitus, autoimmune or connective tissue disorders, or is produced by self-mutilation or may be induced iatrogenically as a result of surgical procedures or anti-cancer therapies.[1] It would be more serious when the body defense is overwhelmed which results in colonization of microorganisms on the injured area and invasion of these microbes or their toxins to the internal layers. Moreover, oral wound may cause pain and discomfort, leaving the patient with a painful chewing function and ultimately a compromised nutrition and a decreased quality of life.[2]

In the past, mucosal wound treatments were focused on palliative and antimicrobial methods.[3] Recently, with the introduction of low-level lasers to medical fields, attentions have been switched towards light-based healing promotion rather than palliation.[4-5] Low-level laser therapy has been touted as a comfortable light-based therapy of wounds.[6] Previous investigations demonstrated that this alternative modality has cellular effects on fibroblast proliferation, collagen production, granulation tissue formation, and modification of the inflammatory response.[7] Since the introduction of photodynamic therapy (PDT) in 1995, laser application has made a more specific and potent form.[8]

In this technique, a dye (photosensitizer) such as methylene blue (MB) with capability of absorbing a specific wavelength is used in target tissue before emitting a laser beam with the same wavelength. So, the dye uses this energy including the excited reactions involved in healing a wound in the desired site.[9] The efficacy of PDT in wound healing is still controversial, probably due to the difference in type of lasers and photosensitizers used in different studies.[10-13]

MB-mediated PDT has shown promising antimicrobial effects in infectious wounds;[14-16] yet, its effects on healing processes are not well-defined.[12, 16-17] Furthermore, previous studies investigated the healing-promoting effect of PDT only on skin tissues, so it is necessary to explore this new technique on mucosa since various tissues response differently to injuries.[18]

The purpose of the present study was to evaluate histological influences of PDT on healing process of experimental oral mucosal wounds in rat model. 

## Materials and Method

All the experiments performed in animals were approved by the Committee for Animal Research Ethics, Torabinejad Dental Research Center, Isfahan University of Medical Sciences. In this study, 48 four-month-old male Wistar rats, weighing approximately 250 g, were recruited. The animals were caged individually and had free access to water and solid food. To begin the intervention, the animals were anesthetized with an intramuscular injection of 10% ketamine considering their weight. The buccal mucosa was selected due to its ease of access.

The surgical procedure consisted of one circular standardized incision performed with a 2-mm diameter punch (size 4) in the left buccal mucosa of each rat. Then, they were randomly divided to 3 groups of 16 rats and received the following treatments; in Control Group (CG), the animals received no treatment and were allowed to heal by secondary intention healing; in Laser Group (LG), animals received laser irradiation on the wound site and in Photosensitizer + Laser Group (PLG), samples received topical MB dye solution (2%) for 5 minutes. It was applied immediately after the wounds were created and was followed by laser irradiation in the same way performed in LG.

The low-intensity laser device used in this study was a continuous mode diode laser (Azor Ltd.; Laser Medical Equipment Moscow, Russia) with 660-nm wavelength and input power of 25 mW. Laser irradiation was performed in a contact mode, for 10 seconds per point with an energy density of 1 J/cm^2^.

Treatment procedure was done in days 1-4 and 6-9 in each two LG and PLG according to standard protocol of laser therapy. After 2, 4, 7, 14-days of interval between the surgical excision and the corresponding interventions, the animals of each group were all sacrificed by using an overdose of anesthetic solution (4 animals in each group in each interval). The lesion was manually delimitated with a draw tool in a way that the tissue specimens contained the whole wound area. The obtained pieces were fixed in 10% formalin for 24 hours. After this stage, paraffin blocks were made and 5-mm thick sections were obtained and stained with Hematoxylin and Eosin (H&E) staining. A qualitative assessment was done according to Shafer histological grading criteria summarized in Table 1.[19]

**Table 1:** Histological grading criteria for healing according to Shafer criteria[19]

**Grade 1 (very light healing)**	**Grade 2 (moderate healing)**	**Grade 3 (advanced healing)**	**Grade 4 (well­-organized)**
Low collagen content, scarce vascularity or low number of capillaries, absence of granulation tissue, abscess formation, necrotic epithelium (Figure 1a)	Moderate collagen content, moderate number of capillaries, onset of granulation tissue formation, epithelial proliferation in the margin of ulcer (Figure 1b)	Abundant collagen content, abundance of capillaries, presence of a well-organized granulation tissue , continuation of epithelialization (Figure 2a and 2b)	Fibrous connective tissue, normal amount of capillaries, absence of granulation tissue, complete ­­­­­­­­­­­­­­epithelialization (Figure 3)

**Figure 1:**
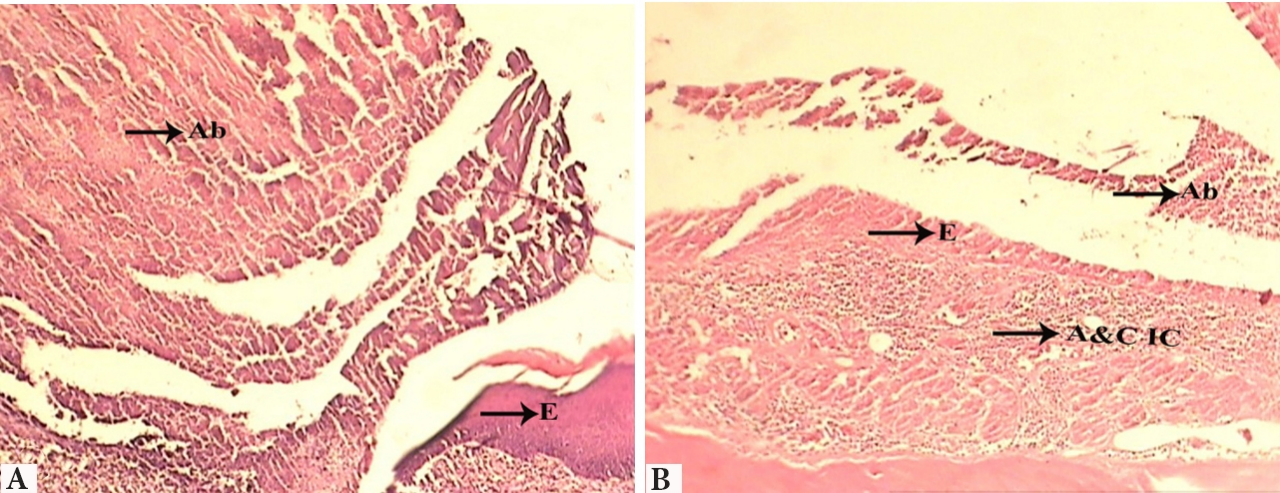
A: Control group (2nd day), Ab: Abscess, E: Epithelium, X100 ; B: Laser group (2nd day), Ab: Abscess, E: Epithelium, A&C IC: Acute and Chronic Inflammatory Cells, X100

**Figure 2:**
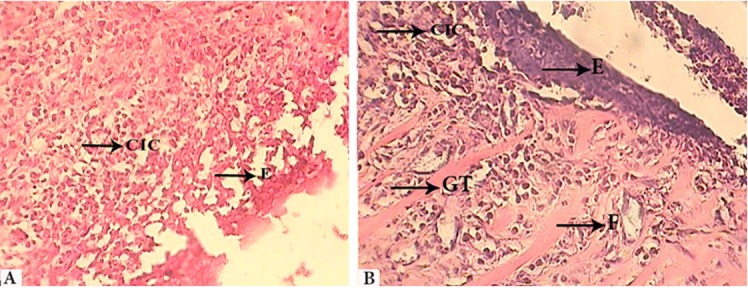
(grade 3): A: Photosensitizer + Laser group (7th day) ; B: Control group, E: Epithelium, CIC: Chronic Inflammatory Cells, GT: Granulation tissue, F: Fibrous tissue. X400

**Figure 3:**
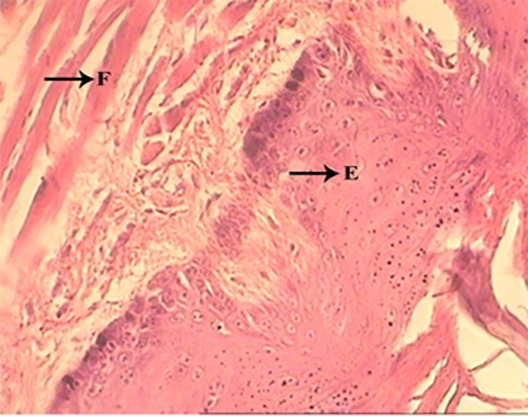
Control group (14st day), E: Epithelium, F: Fibrous tissue, X400

## Results

**Two-day follow-up**

All the wounds showed abscess formation and epithelial necrosis; 50% of animals in PLG and 25% in LG showed grade 2 healing and the rest were in grade 1. All specimens in CG were in grade 1 at this time period. Clinically, all groups had swelling and ulcer.

**Four-day follow-up**

All groups had almost the same histological grade at this time of experiment; however, the tissues were more organized in PLG since grade 3 of wound repair was observed in one of the animals in this group. Proliferation of marginal epithelium of ulcer was initiated. Clinically, all groups had ulcer and decreased swelling.

**Seven-day follow-up**

All animals in LG displayed lower degree of wound healing compared with CG and PLG. More than 50% of animals corresponded to PLG showed grade 3 of healing. This grade was 0% in LG and 100% in CG. More than 50% of the animals in LG were in grade 1. Continuing of epithelialization was observed in all groups with grade 3 of healing. Clinically, swelling was obviated in all groups. In addition all groups had ulcer, although the number of ulcers decreased, particularly in CG.

**Fourteen-day follow-up**

On day 14, both PLG and LG showed a less degree of healing than CG. Epithelialization was complete in CG. Clinically all groups showed healing, although intactness of surface was complete in control group. 

## Discussion

It is believed that the effect of PDT is related to its biostimulatory activity rather than its thermal or chemical actions.[20]

According to literature, applications of PDT in wound healing have not only been for wound healing progression, but also for its proved antimicrobial efficacy.[21-24] On the other hand, this antimicrobial effect can indirectly accelerate the wound healing process.[17] Since infection is a known etiologic factor in delayed healing, some studies have suggested that certain oral bacteria including *S.mutans* and *S.sobrinus* can be efficiently inactivated after application of PDT with dyes such as methylene blue.[11, 25]

However, the efficacy of PDT in wound healing is still controversial. Kubler *et al.* studied the effects of PDT on wound healing of skin lesion in rats and showed that PDT delayed the healing process and decreased the tensile strength as well as the epidermal necrosis of wounds corresponding to this treatment modality.[26] The same results were achieved by the present study which showed a decreased healing grade on day 14 in the PLG, in spite of the neutral or accelerating effect in previous days (2, 4 and 7-day follow-ups) in this group.

In contrast to the present study, Sperandio *et al.* observed the best healing progression in the LG.[12] The present study demonstrated a delayed healing in the LG on days 7 and 14 of the experiment compared with the CG. According to these findings, it is concluded that application of diode laser with the parameters used in this study resulted in a healing inhibitory provocation in delayed phases of healing process for PDT and in earlier phases for laser utilization. Perhaps in this energy density, light absorption occurs in fibroblasts and leads to an inhibitory effect in this type of cells. On the other hand, the innocuous effect of PDT on wound healing process during the first seven days would allow its application on wounds in this limited period to benefit from its antimicrobial effect; since in the preliminary days of an injury, suppression of infection is of paramount importance.[25] It is also important to use the higher energy densities of diode lasers in future experiments.[26]

The use of the same technique in an immunocompromised model is also suggested; as authors believe that in a wound healing process, laser influences the localized immune system and consequently, its efficacy is perhaps achieved only in immunocompromised targets.[27] The authors of the present study also suggest that multiple doses of PDT with various time intervals between doses may speed up the positive effects of this treatment on healing as previously stated by Silva *et al.*[11]

The present study was the first application of PDT in oral mucosal wounds. It was observed that PDT delayed the wound healing in fourteenth days of experiment. This result was in agreement with most previous *in-vitro* studies that indicated a decrease in cell growth or viability.10 Nevertheless, many *in-vivo* investigations reported dissimilar (neutral, decreasing, or accelerating) effects of PDT.[10-12]

Despite the partial similarity of laser parameters and photosensitizer concentration with the present study, Sperandio *et al.*[12] reported a neutral effect of PDT modality on skin wounds. They also suggested that MB could promote a partial or total absorption of the laser light; altering the way the laser interacts with tissue.[12] In this manner, the effects of laser light on tissue could be altered by a possible shielding effect promoted by the amount of dye present inside the wound. The MB is a phenothiazine dye that has been used in medical practice for more than 100 years and was recognized as having very low tissue toxicity. In the present study, the MB could still be seen clinically beneath the crusts of the PLG, even 24 hours after application. Similar clinical evaluation of wounds in both PLG and CG showed no signs of toxic effect from MB. The limitation of the present study was small number of samples that consequently would lead to partial inconclusive results. For further studies, it is suggested to use larger sample size to obtain more precise results. 

## Conclusion

According to findings of the present study, an inhibitory healing effect was seen after 14 days of PDT application. So it is suggested, to use this modality in the earlier stages of wound repair to benefit from antimicrobial effects of photodynamic therapy without any delay in wound healing. 

## References

[1] Squier CA, Kremer MJ (2001). Biology of oral mucosa and esophagus. J Natl Cancer Inst Monogr.

[2] Patton DW, Ali A, Davies R, Fardy MJ (1994). Oral rehabilitation and quality of life following the treatment of oral cancer. Dent Update.

[3] Akopian G, Nunnery SP, Piangenti J, Rankin P, Rinoie C, Lee E (2006). Outcomes of conventional wound treatment in a comprehensive wound center. Am Surg.

[4] Medrado AR, Pugliese LS, Reis SR, Andrade ZA (2003). Influence of low level laser therapy on wound healing and its biological action upon myofibroblasts. Lasers Surg Med.

[5] Basso FG, Pansani TN, Turrioni AP, Bagnato VS, Hebling J, de Souza (2012). In vitro wound healing improvement by low-level laser therapy application in cultured gingival fibroblasts. Int J Dent.

[6] Lucas C, Stanborough RW, Freeman CL, De Haan (2000). Efficacy of low-level laser therapy on wound healing in human subjects: a systematic review. Lasers Med Sci.

[7] Prindeze NJ, Moffatt LT, Shupp JW (2012). Mechanisms of action for light therapy: a review of molecular interactions. Exp Biol Med (Maywood).

[8] Fisher AM, Murphree AL, Gomer CJ (1995). Clinical and preclinical photodynamic therapy. Lasers Surg Med.

[9] Jayasree RS, Gupta AK, Rathinam K, Mohanan PV, Mohanty M (2001). The influence of photodynamic therapy on the wound healing process in rats. J Biomater Appl.

[10] Peplow PV, Chung TY, Baxter GD (2012). Photodynamic modulation of wound healing: a review of human and animal studies. Photomed Laser Surg.

[11] Silva JC, Lacava ZG, Kuckelhaus S, Silva LP, Neto LF, Sauro EE (2004). Evaluation of the use of low level laser and photosensitizer drugs in healing. Lasers Surg Med.

[12] Sperandio FF, Simões A, Aranha AC, Corrêa L, Orsini Machado (2010). Photodynamic therapy mediated by methylene blue dye in wound healing. Photomed Laser Surg.

[13] Peplow PV, Chung TY, Baxter GD (2010). Laser photobiomodulation of wound healing: a review of experimental studies in mouse and rat animal models. Photomed Laser Surg.

[14] Zolfaghari PS, Packer S, Singer M, Nair SP, Bennett J, Street C (2009). In vivo killing of Staphylococcus aureus using a light-activated antimicrobial agent. BMC Microbiol.

[15] Braham P, Herron C, Street C, Darveau R (2009). Antimicrobial photodynamic therapy may promote periodontal healing through multiple mechanisms. J Periodontol.

[16] Kashef N, Esmaeeli Djavid G, Siroosy M, Taghi Khani A, Hesami Zokai F, Fateh M (2011). Photodynamic inactivation of drug-resistant bacteria isolated from diabetic foot ulcers. Iran J Microbiol.

[17] Brown S (2012). Clinical antimicrobial photodynamic therapy: phase II studies in chronic wounds. J Natl Compr Canc Netw.

[18] Szpaderska AM, Zuckerman JD, DiPietro LA (2003). Differential injury responses in oral mucosal and cutaneous wounds. J Dent Res.

[19] Shafer W, Hine M, levy B (1983). A textbook of oral pathology.

[20] Sahu K, Sharma M, Bansal H, Dube A, Gupta PK (2013). Topical photodynamic treatment with poly-L-lysine-chlorin p6 conjugate improves wound healing by reducing hyperinflammatory response in Pseudomonas aeruginosa-infected wounds of mice. Lasers Med Sci.

[21] Demidova TN, Hamblin MR (2004). Photodynamic therapy targeted to pathogens. Int J Immunopathol Pharmacol.

[22] Dai T, Tegos GP, Zhiyentayev T, Mylonakis E, Hamblin MR (2010). Photodynamic therapy for methicillin-resistant Staphylococcus aureus infection in a mouse skin abrasion model. Lasers Surg Med.

[23] Morley S, Griffiths J, Philips G, Moseley H, O'Grady C, Mellish K (2013). Phase IIa randomized, placebo-controlled study of antimicrobial photodynamic therapy in bacterially colonized, chronic leg ulcers and diabetic foot ulcers: a new approach to antimicrobial therapy. Br J Dermatol.

[24] Dai T, Huang YY, Hamblin MR (2009). Photodynamic therapy for localized infections--state of the art. Photodiagnosis Photodyn Ther.

[25] Guo S, Dipietro LA (2010). Factors affecting wound healing. J Dent Res.

[26] Kübler A, Finley RK 3rd, Born IA, Mang TS (1996). Effect of photodynamic therapy on the healing of a rat skin flap and its implication for head and neck reconstructive surgery. Lasers Surg Med.

[27] Yu W, Naim JO, Raymond J, Lanzame MD (1994). The effects of photo-irradiation on the secretion of TGF-b & PDGF from fibroblasts in vitro [abstract 34]. Lasers Surg Med.

